# Modeling of Magnetic Field and Transients in a Novel Permanent Magnet Valve Actuator †

**DOI:** 10.3390/s20092709

**Published:** 2020-05-09

**Authors:** Andrzej Waindok, Bronisław Tomczuk, Dariusz Koteras

**Affiliations:** Department of Electrical Engineering and Mechatronics, Opole University of Technology, PL-45758 Opole, Poland; b.tomczuk@po.edu.pl (B.T.); d.koteras@po.edu.pl (D.K.)

**Keywords:** electromagnetic valve actuator, permanent magnets, finite element analysis, magnetic field integral parameters, simulation of the transients, field-circuit modelling, measurement verification

## Abstract

This paper concerns mathematical modelling of dynamic performances to a new permanent magnet electromagnetic valve actuator (PMEVA). Both static and transient characteristics were simulated by using the finite element method (FEM) and field-circuit approach. The magnetic force values versus the excitation current and the position of the valve actuator movable part have been determined. Our concept of the mover positioning relative to the radial magnets is quite novel. PMEVA parameters are satisfied for implementation in combustion engines. Transients in the device have also been analyzed for no-load and for the nominal burden of the actuator. The indications of the position sensors and the excitation current waves were simulated and measured for the step voltage supply. The calculation results were verified experimentally, and a good conformity has been observed. The advantages of our actuator are simple construction, short time of the switching, the current supplying being needed only at the runner extreme positions, and simple controlling. Additionally, PMEVA design can be extended to support the simultaneous operation of four valves.

## 1. Introduction

Although a trend towards electric car development can be observed, gasoline engines are still the most common drive units in motorization. The increasing demand for gasoline engines’ efficiency and the new stringent regulations for NOx, HC and CO_2_ emissions forces the search for some new modification in existing motors. Many innovations have been introduced in internal combustion engines in recent years. One of the most important approaches is an application of fully controlled valves [1]. There are two main ways to do this. The first one is the use of hydraulic or electro-hydraulic actuators [2,3,4], and the second one can be the application of fully electromagnetic ones [5,6,7,8,9,10,11].

Electromagnetic actuators can be manufactured without applying permanent magnets [5,6,7,8] or with them [9,10,11]. For example, in [5] a numerical analysis of a magnet-less actuator is presented. The influence of the pole shape in this construction has been analyzed. The force calculation has been executed depending on the pole shape. In [6] and [7], the same simple construction has been analyzed. The construction is based only on the electromagnets. The paper [6] focuses on the control method based on energy and force balance in application for the actuator supplied with the 42 V voltage source. In the paper [7], an experimental validation of the construction based on double electromagnets to actuate engine valves is performed. The disadvantage of the actuator is the DC current, which flows permanently in each cycle of operation. A magnet-less solenoid actuator is analyzed in paper [8]. The construction of the actuator is relatively simple, and only its static parameters are given.

In [9] and [10], the constructions of the valve actuator with permanent magnets have been described. In the analysis of this construction, the permanent magnets (PMs) have been taken into account, but the eddy currents in the shorted wire have not been included. However, based on some genetic algorithm, its optimization has been carried out [9]. Short (less than 4 ms) closing time is achieved but it has been obtained for a high supplying voltage of 200 V. Contrary to this achievement, the actuator described in [10] is characterized by long switching time (>20 ms). This paper includes 2D FEM analysis and dynamic coupled mechanical-electrical circuit analysis using circuit blocks. However, no measurement verification is presented. The construction presented in [11] is a bi-stable one. The solid steel mover position is changed by the magnetic field excited in the coils. The permanent magnets are placed on the top and the bottom part of the housing, and only keep the mover in the extreme positions. The construction is quite complicated and those PMs can be damaged during actuator operation.

We present the simulation of a novel permanent magnet electromagnetic valve actuator (PMEVA) operation. Contrary to the electro-hydraulic valve actuators, our construction does not need the hydraulic block and, despite this, it achieves low switching times (5 ms). The obtained values of the runner stroke times are similar to those obtained in [2] and [3]. The presented paper is a substantial extended version of the conference ISEF 2019 presentation [12], and gives more details about the described actuator design.

For all intents and devices, the switching time of an electromagnetic valve depends on the current and supply voltage of its inductor excitation. Due to the application requirements for the combustion engines, the valve actuator was developed as a bi-stable one. This applies two stabile positions of the runner (mover), i.e., the movable part of the actuator. The initial electrodynamic suspension of the mover was excited by permanent magnets, which are radially magnetized, and in this way a simple, compact system for controlling of the valve for combustion engines has been used. Our concept of the mover positioning relative to the radial magnets is quite novel. Thanks to changing the PMs’ location and energy it is possible not only to change the runner range but also its dynamics properties. Applying of permanent magnets improves parameters of the valve actuator compared to construction without PMs [7]. Compared to other permanent magnet structures, the one discussed here is simple in construction and control, and thus more reliable than those described in [9,10,11].

## 2. Physical Model of the Actuator

**Figure 1:**
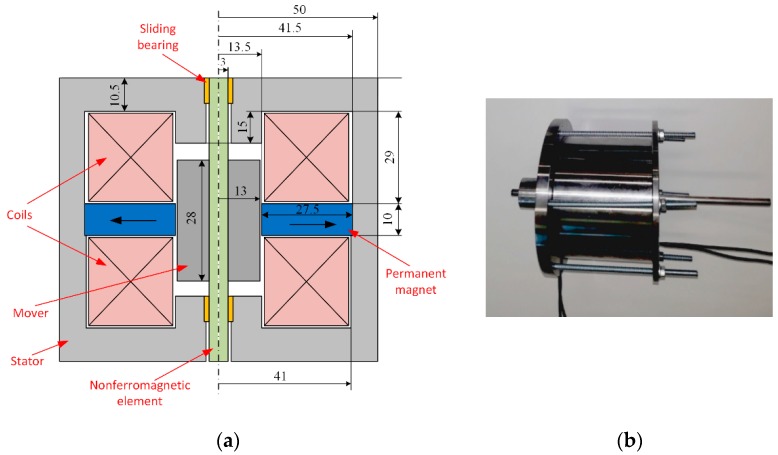
(**a**) Main dimensions of the actuator and (**b**) picture of the prototype.

To evaluate the correctness and helpfulness of the proposed novel construction and calculation method, the measurement verification of characteristics has been carried out for the physical model manufactured at The Department of Electrical Engineering and Mechatronics (DEEM) of Opole University of Technology. A cross section of the axially symmetrical actuator with its main dimensions is given in Figure 1a. Its two identical coils are wounded so that their symmetry axis is in compliance with the actuator axis. The turn number of each one is *N* = 72 and they are connected in series. They are made from copper wire of 2 mm diameter.

The actuator’s outer dimensions were limited by the allowable space (volume) in the combustion engine. Due to a relatively high stroke of the mover (8 mm) the construction is relatively long. However, the use of the Neodymium strong magnets made it possible to reduce the dimensions of the device. The effective NdFeB N35H four magnets, each in the form one-quarter of a ring, were used in the construction. The perpendicular (to the symmetry axis) magnetizing directions have been depicted in Figure 1. Those magnets are characterized by the small value of relative magnetic permeability µ_r_ = 1.053 and high value of the magnetic field coercive force *H_c_* = 880 kA/m. The running rod of the mover was made of non-ferromagnetic material such as stainless steel 304 (also known as 1.4301 steel). Slider sleeve bearings made of phosphor bronze were used. This allowed us to reduce friction, increase structure reliability and to reduce costs. The picture of the prototype PMEVA manufactured in DEEM is presented in Figure 1b.

**Figure 2:**
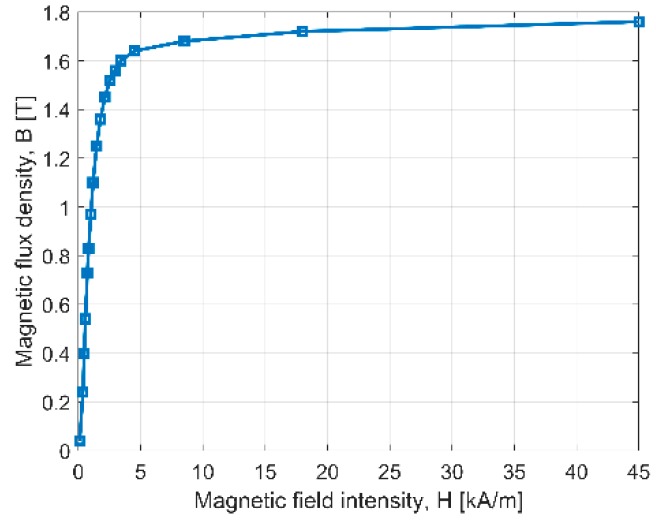
Measured B(H) curve of the steel S355J2H.

The B/H curve of the steel S355J2H used for actuator stator and mover material is not given by the producer. Thus, we have performed the measurements for the open solid sample with using a magnetic yoke [13]. The resulting *B*(*H*) curve of the solid steel (armature material) is presented in Figure 2. It allowed us to use, in calculations, a real nonlinear magnetizing curve of the material.

The dimensions of the actuator’s physical model were measured with the error of 1%. We have included them in the field model. The actuator was mounted on only one valve of the gasoline engine. Due to the presence of the cylinder head, the actuator shaft was longer than it would be in a real operating engine. There is also a possibility to use four movers in one housing to decrease the magnet mass per valve. In such a case, the construction could be mounted above combustion engine cylinders. Thus, the shaft length and mass could be reduced by approx. 30%. Due to the simplicity of the construction, its conservation is relatively easy and inexpensive.

**Figure 3:**
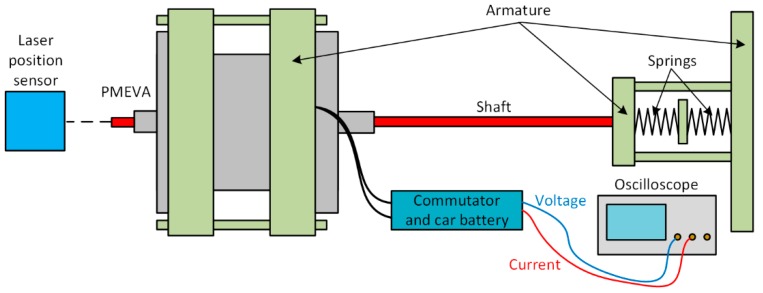
Outline of the measurement stand.

In Figure 3, a simplified outline of the original measurement stand is presented. It was designed for investigation of transients under spring load. To determine the position of the mover, LK-G402 laser sensor was used. The voltage wave was registered directly on the oscilloscope, while the current wave was determined with using LEM/PR30 current transducer. The springs are placed opposite one another, which causes the resulting spring constant to be a sum of the single spring constants. It is also possible to use one spring; however, in such a case, the switching time in one direction increases.

We want to underline advantages of our construction. The excitation field generated by a current in a winding is needed only for switching the extreme positions. Otherwise, in most other constructions the current exists permanently [5,6,7,8]. To obtain high force values (above 400 N), electro-hydraulic construction is used [2,3,4]. Thanks to this, their switching times are relatively short (3–5 ms). However, contrary to our construction, they have relatively significant dimensions and huge hydraulic system for piston drive.

Using the more appropriate wire springs it is possible to reduce the switching time. However, the existing models are characterized by the switching time [9,11], which is comparable with our results. The reduction of the switching time is also possible by means of reducing the mover mass, which is also an advantage of our construction.

## 3. Numerical Modelling of the Magnetic Field

Due to axial symmetry of PMEVA, in our numerical field analysis a 2D finite element method (FEM) was used [14,15,16]. In the first step, the magnetic vector potential (**A**) distribution was determined based on the solution of the nonlinear Poisson’s differential equation:

(1) $$\nabla \times \left(\frac{1}{\mu \left(B\right)}\nabla \times A\right)=J,$$

where ∇−del operator.

Including the cylindrical symmetry, only *J*_φ_ component of the excitation current density exists, and the *A*_φ_ component of the vector potential governs the magnetic field in the calculation domain:

(2) $$\frac{\partial }{\partial r}\left[\frac{1}{\mu \left(B\right)}\left(\frac{\partial A_{\varphi }}{\partial r}+\frac{A_{\varphi }}{r}\right)\right]+\frac{\partial }{\partial z}\left[\frac{1}{\mu \left(B\right)}\frac{\partial A_{\varphi }}{\partial z}\right]=-J_{\varphi }$$

Taking into account the curl of the vector potential (∇×A), the radial and axial component of magnetic flux density vector **B**, can be calculated in the cylindrical system

(3) $$B=\nabla \times A=-\frac{\partial A_{\varphi }}{\partial z}1_{r}+\left(\frac{\partial A_{\varphi }}{\partial r}+\frac{A_{\varphi }}{r}\right)1_{z}$$

The magnetic force (**F**) has been determined using Maxwell’s stress tensor method [16]

(4) $$F=\oint_{\Gamma }\;\left[\begin{array}{cc} \mu \left(B\right)\left(H_{r}^{2}-\frac{1}{2}H^{2}\right) & \mu \left(B\right)H_{r}H_{z}\\ \mu \left(B\right)H_{r}H_{z} & \mu \left(B\right)\left(H_{z}^{2}-\frac{1}{2}H^{2}\right) \end{array}\right]\cdot d\Gamma ,$$

where µ is magnetic permeability and **Γ** is the contour of the ferromagnetic runner.

The dynamic inductance *L_d_* of the excitation coil was calculated from the current derivative of the magnetic flux which is linked with the coil turns, and the electromotive force (EMF) can be calculated from the position derivative [17]

(5) $$L_{d}=\frac{\partial \Psi }{\partial i},\;EMF=\frac{\partial \Psi }{\partial z}$$

**Figure 4:**
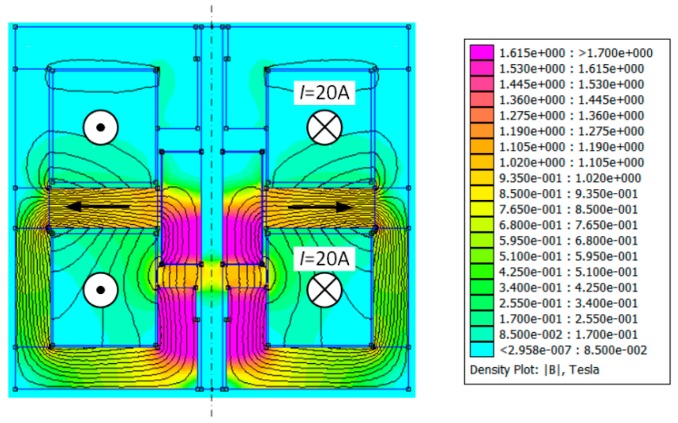
Magnetic flux density distribution in the longitudinal cross section for *z* = 0 and *I* = 20 A.

**Figure 5:**
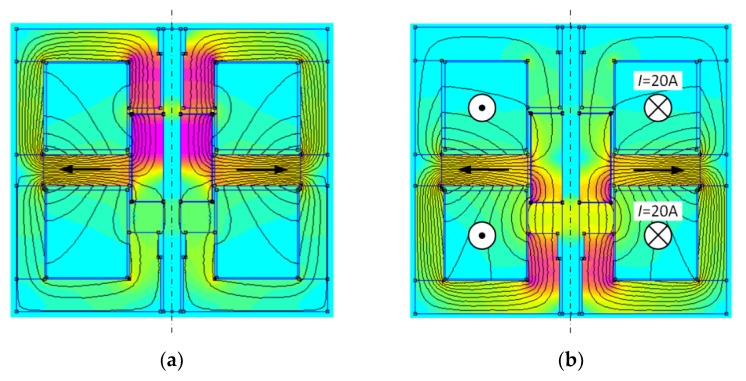
Magnetic flux density distribution in the actuator cross section: (**a**) for *z* = 4 mm and *I* = 0 A, (**b**) for *z* = 4 mm and *I* = 20 A.

Using the presented model, calculations of the magnetic flux density distribution were made for different mover positions and for various values of the excitation current intensities. Some of the results are shown in Figure 4 and Figure 5. For the neutral position of the runner and zero-value of the current intensity in the coil, the magnetic force vanishes. In order to generate the force, the position (“*z*”- coordinate) of the runner should be changed or the excitation current should be supplied. This case is depicted in Figure 4, where the magnetic flux arisen by the ampere-turns in the windings increases the magnetic flux in the lower part of the actuator parallel to a reduction in it at the top part of the device. Thus, in spite of the neutral position of the runner, the magnetic thrust arises and is directed down.

In the case of no-current excitation and maximum mover stroke, the force arising from the permanent magnet field keeps the mover in the position presented in Figure 5a. In the extreme position of the runner, its partial saturation occurs, which results in pushing the stream out the saturated part, which is called fringing flux effect. However, this does not bother the runner and, on the contrary, supports the magnetic force of the movable part for its extreme position.

To change the direction of the magnetic force, the PM field needs to be reduced by the flux excited in the coils. For such a case the force acting on the runner’s top part is decreasing, while the force influenced its bottom part is significantly increasing. In Figure 5b, we present the field distribution in such an instance. The additional spring acting on the mover can help the mover to change the position to the opposite one, which is not presented in the figure.

**Figure 6:**
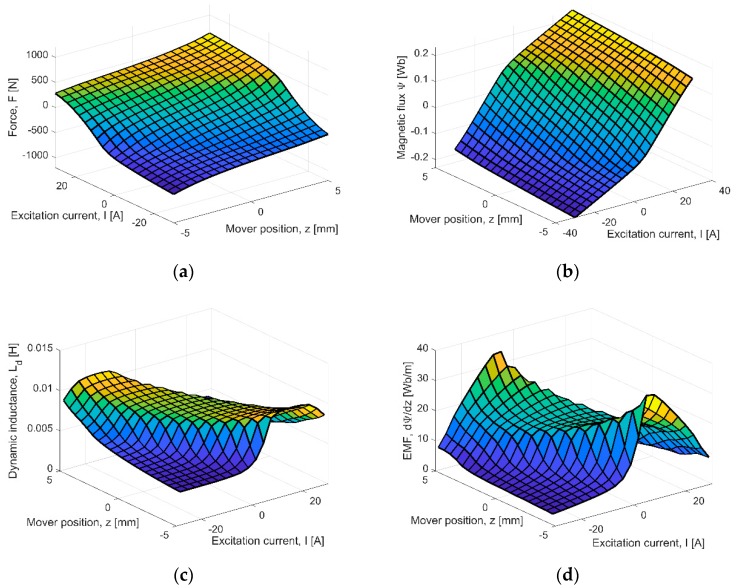
Integral parameters verso mover position *z* and excitation current value *I*: (**a**) thrust, (**b**) magnetic flux, (**c**) dynamic inductance, (**d**) electromotive force (EMF).

The magnetic force (thrust), magnetic flux and dynamic inductance of the excitation coils are called the integral parameters of the electromagnetic field. Using the model presented above, the calculations of the mentioned quantities, as the functions of the runner position (“*z*”- coordinate) and excitation current, were carried out. Results are given in Figure 6.

The characteristics of the thrust for the PMEVA prototype (Figure 6a) are consistent with the physical phenomena of electromagnetism. The force is highest for the extreme mover positions (±4 mm) and for maximal values of the excitation current. It should be added that it is possible to change the direction of the force by changing the direction of the excitation current. Due to the magnetic saturation effect, it is not advisable to force the current intensity value more than 25 A. This is pointless because, for the current-less excitation, the force in the extreme positions of the runner is highest (Figure 6a) and reaches more than 320 N. This is also favorable in controlling the operation of the device, where the springs are used. This is due to the highest force arising from the PMs, which is higher than the force of the spring. The correct selection of the spring characteristic allows the excitation current to switch off at extreme positions of the movable part.

The characteristic of magnetic flux, linked with the winding, changes smoothly verso the mover position and the excitation current values (Figure 6b). For the highest current values and the extreme mover positions, a saturation effect is observed. Due to the smoothness of the flux characteristic, it is simple to calculate its derivatives.

The dynamic inductance *L_d_* of the excitation coil is also the integral parameter of electromagnetic field. Its calculation value, as the function of the runner position and various excitation current values, is given in Figure 6c. The magnetic saturation effect is observed in the graph. For the saturated steel armature, the inductance value can be less twice than the value obtained for non-saturated material. The highest value of the inductance is observed for small values of the current intensity and for the outmost position of the actuator mover.

The position derivative of the flux is called electromotive force (EMF). The characteristic of EMF (Figure 6d) is similar to the dynamic inductance. The highest values are observed for no-current state and for the outmost mover positions. The increasing of the current value causes a reducion of EMF.

## 4. Numerical Modelling of Transients

In order to decrease the calculation time, a field-circuit model based on the stored values of integral parameters, obtained from the field analysis (Section 3), was used. The setup presented in Figure 1 could be described by two variables, i.e., the electric charge *Q*(*t*) and the mover position *z*(*t*). Some additional parameters need to be used in order to describe the system dynamics: *k* – spring constant, *D* – friction coefficient, *m* – mass of the mover, *v* – mover velocity, *u* – supplying voltage, *R* –resistance of the coils and wires. From the field model, the force *F* and magnetic flux linkage Ψ values verso current value and mover position are obtained (Section 3).

In order to obtain mathematical model for transients, an Euler-Lagrange method was used [18,19,20]. The vector of unknown variables for the circuit model is:

(6) $$q=\left[i,z\right]=\left[\dot{Q},z\right]$$

Coenergy of the system is described with using the following expression [14]:

(7) $$T^{\prime }=\frac{1}{2}m{\dot{z}}^{2}+\overset{\dot{Q}}{\underset{0}{\int }}\Psi \left(\dot{Q},z\right)d\dot{Q}$$

The potential energy is stored in springs and is equal to:

(8) $$U=\frac{1}{2}kz^{2}$$

Lagrange’s function takes the following form

(9) $$L=T^{\prime }-U=T^{\prime }=\frac{1}{2}m{\dot{z}}^{2}+\overset{\dot{Q}}{\underset{0}{\int }}\Psi \left(\dot{Q},z\right)d\dot{Q}-\frac{1}{2}kz^{2}$$

In order to formulate Euler-Lagrange equations, a virtual work method is used. The energy increase of the system is equal to:

(10) $$\delta A=\tilde{P_{Q}}\delta Q+\tilde{P_{z}}\delta z=\left(u-Ri\right)\delta Q+\left(-D\dot{z}\right)\delta z$$

For each independent variable one ordinary differential equation, describing the transient behavior of the system, is obtained:

(11) $$\frac{d}{dt}\left(\frac{\partial L}{\partial \dot{z}}\right)-\frac{\partial L}{\partial z}=\tilde{P_{z}},\frac{d}{dt}\left(\frac{\partial L}{\partial i}\right)-\frac{\partial L}{\partial Q}=\tilde{P_{Q}}$$

Substituting Equations (8) and (9), the following system of ordinary differential equations is derived:

(12) $$m\ddot{z}-\partial \left(\int_{0}^{i}\Psi \left(i,z\right)di\right)/\partial z+kz=-D\dot{z}$$

(13) $$\frac{d}{dt}\left(\partial \left(\int_{0}^{i}\Psi \left(i,z\right)di\right)/\partial i\right)=u-Ri$$

The linkage flux Ψ is a function of two variables. Thus, its time derivative is described by the expression

(14) $$\frac{d\Psi \left(i,z\right)}{dt}=\frac{\partial \Psi \left(i,z\right)}{\partial i}\cdot \frac{di}{dt}+\frac{\partial \Psi \left(i,z\right)}{\partial z}\cdot \frac{dz}{dt}$$

The current derivative of the flux Ψ is usually denoted as dynamic inductance *L_d_* (Equation (5)). Including a coil resistance *R* and supplying voltage value *u*, the following expression could be written

(15) $$L_{d}\left(i,z\right)\cdot \frac{di}{dt}+\frac{\partial \Psi \left(i,z\right)}{\partial z}\cdot v=u-Ri$$

In the second part of the equation, a position derivative of the flux linkage occurs. It is an electromotive force (EMF) induced in the coils under runner movement.

Finally, the following system of the ordinary differential equations was obtained and included in our field-circuit model:

(16) $$\left[\begin{array}{c} L_{d}\left(i,z\right)\frac{di}{dt}\\ m\frac{dv}{dt}\\ \frac{dz}{dt} \end{array}\right]=\left[\begin{array}{c} u\\ F\left(i,z\right)\\ 0 \end{array}\right]+\left[\begin{array}{ccc} -R & -\frac{\partial \Psi \left(i,z\right)}{\partial z} & 0\\ 0 & -D & -k\\ 0 & 1 & 0 \end{array}\right]\left[\begin{array}{c} i\\ v\\ z \end{array}\right]$$

**Figure 7:**
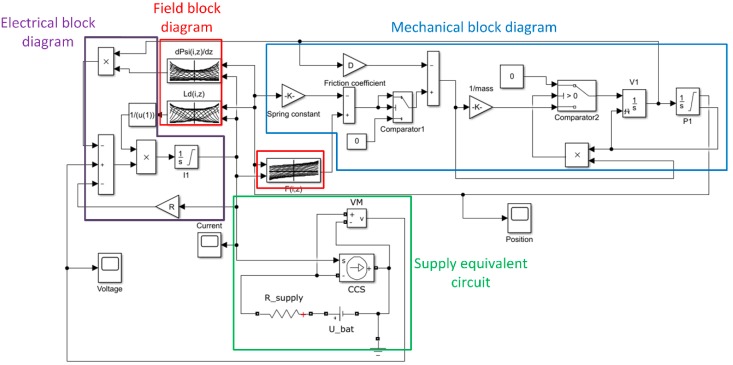
Schematic view of the analyzed dynamic system.

The simplified block diagram including the time derivatives and the equations set, which has been implemented in Matlab/Simulink software, is presented in Figure 7. The diagram was divided into four blocks. In the electrical block, the winding resistance is defined (*R* = 0.292 Ω) and the signals from field block and supply equivalent circuit are collected. In this block, the excitation current value is calculated. In the supply equivalent circuit, the car battery parameters are defined (*R_s_* = 0.078 Ω, *E* = 12.4 V). In the field block diagram, the characteristics given in Figure 6 are included in the form of *Look-up tables*. They are connected with the electrical and mechanical blocks. In the mechanical block the following parameters are defined: spring constant *k*, mover mass *m*, friction coefficient *D*. The values of these parameters were given in Table 1 (Section 5). Additionally, limitations of the movement and interaction between spring and electromagnetic force were included. To take into account the movement ends, two comparators have been modeled in the diagram above.

The existing actuator simulation models are characterized by different times of execution. For example, the solution of elliptic-parabolic partial differential equations (PDE), using 2D FEM analysis (with Maxwell package), took a relatively long time (several minutes) [10,19]. To perform the analysis, our field-circuit model is characterized by a relatively small execution time—we executed our analysis in a few seconds. There are faster models based on the circuit theory, but they can be applied only for relatively simple geometries [2,3,6].

## 5. Measurement Verification of the Calculation Results

### 5.1. Integral Parameters of the Magnetic Field

**Figure 8:**
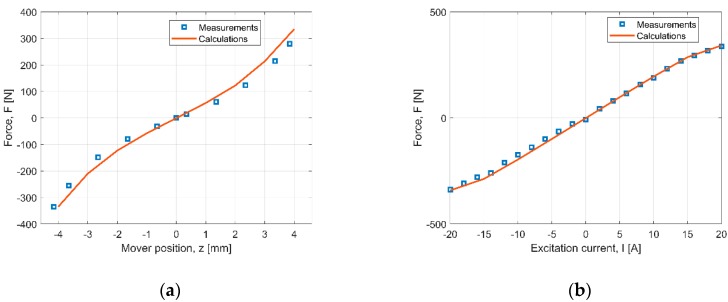
Force verso: (**a**) mover position for *I* = 0, (**b**) current value for *z* = 0.

In order to validate our mathematical model, the measurement verification was undertaken. The force values graph vs. the “*z*” coordinate of the mover position, for current-less excitation (*I* = 0), is given in Figure 8a, while those quantity values, at the neutral runner position, are given in Figure 8b. In the former figure, the force changes exponentially as the air gap decreases (Figure 1) between the mover and stator of the actuator. From the dynamics point of view, it is favorable because the magnetic thrust compensates for the spring forces.

For the neutral position of the runner, when the excitation current intensity magnitude is less than 15 A, the force changes nearly linearly with the quantitative change of the current intensity (Figure 8b). For current intensity magnitude above 15 A, the magnetic circuit saturation effect can be visible. The force increases in value parallel to the coordinate “*z”* increasing up to 340 N. For *I* = 20 A, the force value is 335 N.

In order to compare the measurement and calculation results, a normalized root mean square error (NRMSE) was used:

(17) $$NRMSE=\frac{\sqrt{\frac{1}{N}\sum_{i=0}^{N}{\left(y_{i}^{meas}-y_{i}^{calc}\right)}^{2}}}{max(y_{meas})-\min(y_{meas})}\cdot 100\;\%$$

where: *N*—number of measurement points, $y_{i}^{meas}$—measured value in *i*-th point, $y_{i}^{calc}$—calculated value in *i*-th point.

Our calculation model gives relatively small errors, which are shown in Table 1. In case of the force vs. the mover position *F*(*z*), we obtained 3.16 %. For the characteristic force verso the current value, the error was smaller (1.73 %).

The value of the dynamic inductance was measured and calculated for neutral mover position. The measured value was *L_d_* = 7.66 mH, while the calculated one was *L_d_* = 8.8 mH. The discrepancy in the given values is due to slight simplifications in the mathematical modeling. However, it is probable that a larger error arose in inductance measuring because, due to the magnetic circuit from solid steel, the response to the voltage jump was measured.

### 5.2. The Transients in the Field-Circuit Model

Two different transients were tested. In the first one, the no-load state was investigated, while in the second one the additional springs were employed and the load state was thus analyzed. A step voltage change was assumed. Values of the supplying voltage changed abruptly from 0 to 12.4 V. The parameters which were assumed for the field-circuit model are given in Table 2. The movable element mass “*m*” and the friction constant “*D*” were determined experimentally. The given resistance value “*R + R_s_*” includes the windings’ resistances and internal resistance of the supplying circuit. The spring constant value “*k*” has also been measured.

**Figure 9:**
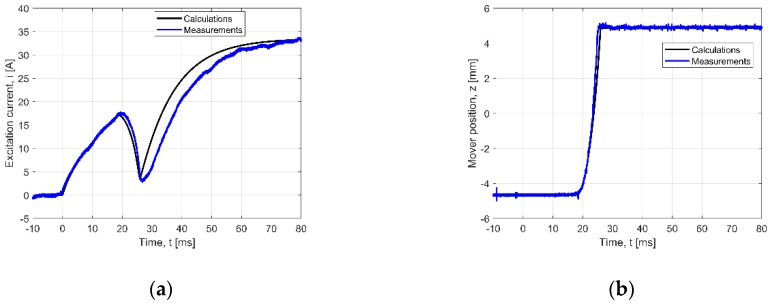
Verified transients for no-load state: (**a**) current wave, (**b**) position wave.

In Figure 9, the results of the measurement verification for the no-loaded actuator are shown. In the case of the current wave (Figure 9a) the generation effect of the electromotive force, due to the mover velocity, is visible. In the first part of the wave, the current increases exponentially to the maximal value. Then, the current decreases to the point where the runner occupies the edge position. After starting the movement of the runner, the EMV increases and the current intensity values decrease. After the movement stops, the current increases exponentially. The movement time between the extreme positions of the runner lasted ca. 9 ms, (Figure 9b). The differences between measurement and calculation values of the current wave are visible after the mover stops for the moment *t* = 27 ms (Figure 9a).

**Figure 10:**
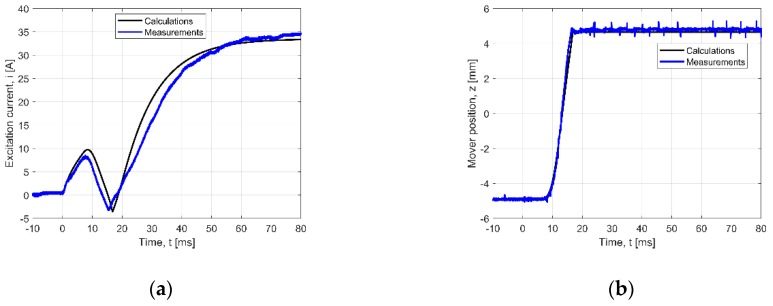
Verified transients for loaded state (springs added): (**a**) current wave, (**b**) position wave

For comparison, Figure 10 shows the transients for the actuator loaded with springs. It shows the excitation current intensity values and the coordinates “*z*” of the mover transfer versus time. The calculated and measured curves for the loaded actuator can be compared with those from Figure 9. Compared with the results obtained for the no-load state, the adding of the springs shortens the movement time. This is due to the fact that the springs help the mover come back. Thus, for the invented construction of the actuator, the use of springs improves the dynamic performance (reaction time) of the PMEVA.

**Figure 11:**
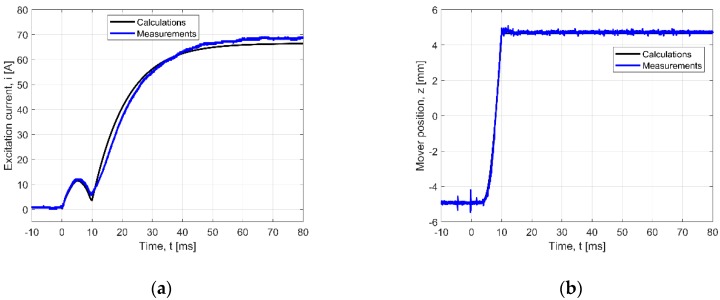
Verified transients for loaded state (springs added) and for increased voltage (*U* = 25 V): (**a**) current wave, (**b**) position wave.

In order to study the influence of the supply voltage on the dynamic properties of the PMEVA, the measurements and calculations for higher voltage values (than 12.4 V) were carried out, (Figure 11). The values varied abruptly from zero to 25 volts (*R_s_* = 0.084 Ω). For the position wave, a very good agreement between measurement and calculation values was obtained (Table 3). For this higher voltage, the current value increased nearly two-fold and the forward and back runner stroke times decreased approximately two-fold (to 5 ms). The higher voltage resulted in higher values of magnetic force. Therefore, the above-described simplifications of the computational model and neglected phenomena have less impact on the analysis results. The calculated and measured curves almost coincide (Figure 11b). This is due to that the calculation and measurement errors have reduced each other.

The calculations errors for no-loaded actuator arise due to some simplifications in the mathematical model. For example, the inductance of the cables connecting the supplying voltage source with the actuator was not taken into account. The stochastic nature of the friction force is also not included. Considering the relatively low electrical conductivity of PMs and the relatively large air gaps in the magnetic circuit of the actuator, the eddy currents were omitted. The currents in the moving part, by the magnetic flux changes and the runner movement, can reduce the thrust values. Moreover, the air gap in the extreme position of the real runner may be slightly different than with the geometry of the calculation model. According to the simplifications, the absolute values of the force were slightly greater than the measured ones. Moreover, the current intensity values, after the mover was stopped were greater than the real ones, and the measured runner time was slightly shorter.

In Table 3 the relative errors for the waves of current and mover position are given. For the characteristic *i*(*t*), in the case of supplying with the relatively low voltage (*U* = 12.4 V), NRMSE exceeds 8% (case 1), but after applying the wire springs it decreases to 5.9% (case 3). For higher voltage value (*U* = 24 V), the currents are much higher and the error is reduced to 2.06% (case 5). Similarly, it is observed for *z*(*t*) wave that increasing the supply voltage causes decreasing NRMSE values. This proves the correctness of our field-circuit model.

## 6. Conclusions

There are some differences between calculation and measurement current waves. The numerical model, compared to the physical object, is characterized by a slightly higher movement time for the mechanical part. In case of the mover relocation (position) waves, the discrepancy of the parameters for the mathematical model and the physical object are relatively small. The differences between the calculation and measurement results are due to: measurement errors, the simplifications assumed for the mathematical model, discretization errors due to small differences in the geometries of the calculation model and the manufactured physical object. Moreover, the measurement system demands a relatively long coupling between mover and springs which caused the movement mass to increase. There is a possibility to reduce it in the real operation system by cooperation with a combustion engine. In such a system the dynamic properties of the EVA device could be improved. For example, the switching time is going to be shorter.

Higher discrepancies are observed in the case of current waves. They are mostly due to small differences in geometry of the physical and numerical models. Ignoring the supplying wires inductances, and the stochastic nature of the friction force have introduced some small errors (Figure 9 and Figure 10, Table 3). The differences between the measurement and calculation results of excitation currents are smaller for higher supplying voltages (Figure 11, Table 3), and a good conformity between experiment and calculations is observed. It should be mentioned that auto manufactures suggest a battery with higher voltage (24 V or even 48 V).

The use of additional springs improves the dynamic properties of the PMEMV, but in order to decrease the switching time, a higher supply voltage should be implemented. The higher voltage value affects the thrust force, and the movement velocity increases. In order to better understand the behavior of the electromagnetic system it is valuable to calculate the voltage induced by the armature movement and include the saturation of the magnetic parts of the circuit.

There are also possible simulations of other applications of the actuator with our model. These are a mixed system with hydraulic valve drive, the source of vibration, impact hammer, simplified version of a piston drive, some devices for fatigue testing of materials. For example, the electromagnetic actuator, which is used as the electric load simulator of the exhaust valve [21], can be simulated with our method. Similarly, the actuator for the gas exchange valve, which is presented in the paper [22], could also be calculated using the field-circuit method described in this paper. Thus, the presented mathematical model allows us to include the different physical phenomena by adding some equations into the field-circuit calculations.

The research into the developed construction shows considerable potential usefulness in the invented device. Its dynamic properties are satisfactory when it comes to implementing it to control a valve’s position in an internal combustion engine. The force value reaches several hundreds of Newtons for the relatively small movable mass (ca. 200 g, including the valve). The main advantage of the investigated actuator is the simple construction and the effective controlling. The implementation of electromagnetic actuators for valves could improve the combustion engine parameters and decrease the fuel consumption.

## 7. Patents

Tomczuk, B.; Waindok, A.; Mamala J. *Ferrodynamical actuator with permanent magnets*, registration no. P.430921 in a Polish Patent Office, 20 August 2019.

## Figures and Tables

**Table 1:** Normalized root mean square error (NRMSE) value for characteristics given in Figure 8 for the number of points *N* = 9.

Characteristic	NRMSE [%]
***F*** **(*z*)**	3.16
***F*** **(*I*)**	1.73

**Table 2:** Parameters of the transient model.

Parameter	m [kg]	D [Ns/m]	R + R_s_ [Ω]	k [N/mm]
**No-load state**	0.153	100	0.370	0
**Loaded actuator**	0.203	50	0.370	46.6

**Table 3:** NRMSE value for transients given in Figure 9, Figure 10 and Figure 11 (*N* = 1600).

Case Number	Performance Graph	Wave	NRMSE [%]
1	Figure 9a	*i*(*t*) – no springs, *U* = 12.4 V	8.18
2	Figure 9b	*z*(*t*) – no springs, *U* = 12.4 V	2.68
3	Figure 10a	*i*(*t*) – *U* = 12.4 V	5.90
4	Figure 10b	*z*(*t*) – *U* = 12.4 V	3.43
5	Figure 11a	*i*(*t*) – *U* = 25 V	2.06
6	Figure 11b	*z*(*t*) – *U* = 25 V	0.93
